# Clinical Performance Status and Technical Factors Affecting Outcomes from Percutaneous Transhepatic Biliary Interventions; A Multicentre, Prospective, Observational Cohort Study

**DOI:** 10.1007/s00270-021-02888-0

**Published:** 2021-07-12

**Authors:** Gregory C. Makris, Andrew C. Macdonald, Kader Allouni, Hannah Corrigall, Charles R. Tapping, Jane Philips Hughes, Suzie Anthony, Phil Boardman, Rafiudin Patel, Andrew Wigham, Mohammad Ali Husainy, Teik Choon See, James Cast, Simon Olliff, Simon Travis, Hans-Ulrich Laasch, Colin Nice, Raman Uberoi

**Affiliations:** 1 Vascular and Interventional Radiology Department, Guy’s and St Thomas’ Hospital, NHS Foundation Trust, London, UK; 2 Alfa Institute of Biomedical Sciences, Neapoleos 9, Marousi, Athens Greece; 3 Department of Interventional Radiology, Oxford University Hospitals NHS Foundation Trust, oxford, UK; 4 University Hospitals North Midlands NHS Foundation Trust, Stoke-on-Trent, UK; 5 Cambridge University Hospitals NHS Foundation Trust, Cambridge, UK; 6 Hull University Teaching Hospitals NHS Foundation Trust, Hull, UK; 7 University Hospitals Birmingham NHS Foundation Trust, Birmingham, UK; 8 Nottingham University Hospitals NHS Foundation Trust, Nottingham, UK; 9 The Christie NHS Foundation Trust, Manchester, UK; 10 Newcastle Upon Tyne Hospitals NHS Foundation Trust, Newcastle upon Tyne, UK

**Keywords:** Percutaneous, Biliary, Drainage, Stenting

## Abstract

**Purpose:**

The purpose of this study was to evaluate the predictive value of a ‘Modified Karnofsky Scoring System’ on outcomes and provide real-world data regarding the UK practice of biliary interventions.

**Materials and Methods:**

A prospective multi-centred cohort study was performed. The pre-procedure modified Karnofsky score, the incidence of sepsis, complications, biochemical improvement and mortality were recorded out to 30 days post procedure.

**Results:**

A total of 292 patients (248 with malignant lesions) were suitable for inclusion in the study. The overall 7 and 30 day mortality was 3.1% and 16.1%, respectively. The 30 day sepsis rate was 10.3%. In the modified Karnofsky ‘high risk’ group the 7 day mortality was 9.7% versus 0% for the ‘low risk’ group (*p* = 0.002), whereas the 30 day mortality was 28.8% versus 13.3% (*p* = 0.003). The incidence of sepsis at 30 days was 19% in the high risk group versus 3.3% at the low risk group (*p* = 0.001)

**Conclusion:**

Percutaneous biliary interventions in the UK are safe and effective. Scoring systems such as the Karnofsky or the modified Karnofsky score hold promise in allowing us to identify high risk groups that will need more careful consideration and enhanced patient informed consent but further research with larger studies is warranted in order to identify their true impact on patient selection and outcomes post biliary interventions.

## Introduction

Percutaneous Transhepatic Biliary Drainage (PTBD), with or without stent insertion, is a valuable technique in the management of biliary obstruction. External drainage or internal drainage via a stent can relieve symptoms and improve serum biochemistry. This can optimise the clinical condition of patients requiring surgical resection or palliative treatements, and improve their quality of life [1–3].

PTBD is an invasive procedure and has associated complications, including bleeding, infection and bile leakage. The British Society of Interventional Radiology Biliary Drainage and Stent Audit Report 2009 demonstrated a 7.9% rate of major complications, a 26% rate of minor complications and an in hospital mortality of 19.8% [4]. The high associated mortality and complication rate indicate a need to identify pre-procedural predictive factors that will enable better patient selection.

The aim of this study was to evaluate a modified functional scoring system (Modified Karnofsky score) in a “real-world data” setting, potentially allowing us to support measures to improve patient outcomes and advise patients appropriately of their individual procedural risks.

## Methods

### Study Design and Objectives of the Study

This was a multicentre, prospective, observational cohort study that ran over a four year period at eight UK sites. The primary objective was to assess if the ‘Modified Karnofsky Scoring System’ utilising performance status scores, co-morbidities and biochemical/haematological markers could gauge the risk of morbidity or mortality from PTBD. The secondary objectives were to assess if the choice between primary drain/stent placement and procedure staging may also affect outcomes.

### Study Population

The study prospectively recruited participants over 18 years of age, undergoing PTBD ± stenting for benign or malignant causes.

### Interventions

As this was an observational study it was at the treating physicians’ discretion to perform stent or drain insertion and also regarding staging of the procedures. The treatments were not affected or dictated by the study protocol.

### Study Assessments

The study was collecting data available in the patient notes and on the electronic patient records and a standardised proforma was completed prior to the procedure. The following were assessed at baseline: demographics; type of stricture (benign/malignant obstruction) and site of obstruction according to Bismuth classification [5]; current treatments; previous medical history; performance status (Karnofsky score); presence of ascites and biochemical/haematological markers (bilirubin, albumin, haemoglobin, platelets, clotting profile and white cell count).

The incidence of sepsis was assessed at 7 and 30 days. Sepsis was defined by meeting the SIRS (systemic inflammatory response) criteria in the presence of suspected infection. The SIRS criteria required at least two of: temperature > 38 or < 36 °C, tachycardia > 90 beats/minute, serum white cell count > 12 or < 4 × 10^9^/L. The level of bilirubin and symptoms were assessed at 7 and 30 days and the responsible physician had to decide if the symptoms were stable, worse or better. The same applied to their bilirubin levels. If the investigators felt that the change in the levels was clinically significant they were asked to report it as such. No cut off values were provided. All-cause mortality was recorded at 7 and 30 days. Immediate post-operative complications were also documented (Table 1).

**Table 1:** Karnofsky Score

Karnofsky Performance Status Score	
Function	Score (%)
Normal, no complaints, no signs of disease	100
Capable of normal activity, few symptoms or signs of disease	90
Normal activity with some difficulty, some symptoms or signs	80
Caring for self, not capable of normal activity or work	70
Requiring some help, can take care of most personal requirements	60
Requires help often, requires frequent medical care	50
Disabled, requires special care and help	40
Severely disabled, hospital admission indicated but no risk of death	30
Very ill, urgently requiring admission, requires supportive measures or treatment	20
Moribund, rapidly progressive fatal disease processes	10
Death	0

### Pre-Procedural Scoring

By taking into account the success of previous studies [4, 6–9] and utilising the predictive markers seen to most strongly correlate with outcome, a novel scoring system was produced (Table 2) (Appendix for CRF). The scoring system developed is the “Modified Karnofsky” score which had previously been piloted on 50 consecutive patients. This is a combination of the Karnofsky performance [10] score with biochemical markers including albumin (g/l), prothrombin time (s) (INR), presence of ascites, haemoglobin (g/dL), bilirubin (mmol/L) and white cell count (× 10^9^/L). Table 2 shows how different values of the previously mentioned markers were assigned points, the sum of which could be grouped into either a low risk group (0–4 points) or a high risk group (≥ 5 points). The classifications thresholds were extracted from the previous pilot study and after consensus was achieved between the members of the steering committee of the study (GM, AM, RU).

**Table 2:** Proposed pre-procedural risk for PTC procedures

Modified Karnofski score			
Outcomes:	Points		
Measure:	0	1	2
Albumin (g/l)	> 35	28–35	< 28
Prothrombin Time (s) (INR)	≤ 16 (< 1.7)	17–20 (1.7–2.3)	> 20 (> 2.3)
Ascites	None	Mild	Moderate to severe
Haemoglobin (g/dL)	≥ 11	< 11—10	< 10
Bilirubin (mmol/L)	< 100	100–300	> 300
White Cell Count (× 10^9^/L)	< 11	11—14	> 14
Karnofsky Score* (%)	≥ 80	60–79	> 60
*Total score*:			
Risk Group:	Low Risk (0–4 points)		High Risk (5 + points)

### Definition End of the Study

The end of study was 30 days following recruitment of the last patient.

### Ethics and Sponsoring

The study was approved by the Research and Development committee of the Oxford University Hospitals NHS Foundation Trust and the National Research and Ethics Service.

The study was supported by an educational grant by the British Society of Interventional Radiology.

### Statistical Analysis

Post interventional complication rates, 7 and 30 day morbidity and mortality as well as incidence of sepsis and resolution of symptoms were correlated with pre-interventional factors such as co-morbidities; namely hypertension (HTN), ischaemic heart disease (IHD), insulin dependent diabetes mellitus (IDDM), presence of ascites, disorders of coagulation and patients on anticoagulant therapy. Outcomes were also measured against performance scoring systems, namely the Karnofsky score, and the ‘modified Karnofsky score’.

Data were analysed using SPSS (IBM). A *p* value of < 0.05 was interpreted to denote statistical significance with a Chi-squared analysis performed to compare outcomes of staged versus primary stenting, high versus low risk groups and stent versus drain only groups. For complications, Fisher’s exact test was applied to compare values in the different groups. Receiver operator curves were used to compare the Karnofsky with the modified Karnofsky score. Binary regression analysis was performed for categorical data to assess if any factors were specifically associated with the incidence of overall 30 day mortality and incidence of sepsis 30 days post operatively.

## Results

### Demographics

From 308 identified patients, 292 were included in the study. The excluded patients had either withdrawn from the study or had endoscopic treatment instead. The mean age was 72.3 (SD: 4.8) and 55.5% of patients were male. The performance status of our cohort, (Karnofsky score) was > 80 in 134 out of 292 patients (45.9%), 60–79 in 112/292 (38.4%) and < 60 in 44/292 (15.1%). Only 14% of cases were treated with chemotherapy and 15.8% were receiving anti-platelets. The following comorbidities were present in our cohort: hypertension (28.8%), heart disease (8.8%), diabetes (5.8%) and coagulopathy (9.2%).

### Technical Information and Overall Outcomes

The majority of the cases (84.9% − 248/292) presented with malignant obstruction and the remaining had benign causes (biliary stones, post-operative strictures and pancreatitis complications were the most common). For the malignant lesions the level of the obstruction according to the Bismuth classification was as follows: type I 7/231 (3%), type II 40/231 (13.7%,) type IIIa-b 26/231 (8.9%), type IVa-b 26/231 (8.9%) and distal type 132/231 (45.2%).

Biliary stents were used in 57.2% (171/292) of cases. 87.1% of these stents (149/171) were metal stents, 11 were plastic and there was one biodegradable stent. The majority of the stents were inserted as a one-stage procedure (120/171–70.1%), whereas 51 out of 171 were performed as a two-stage procedure. Most of the procedures were performed with right sided access (154/205–75.1%) and only 5.4% with bilateral access (11/205). The vast majority of the procedures took place under local anaesthetic and conscious sedation (254/292–86%) and all had preoperative antibiotics.

The technical success, defined as successful drainage, was 95.2% (278/292) while symptoms at day 7 had improved in 44.2% and in a further 34.2% of the patients at day 30. The 30 day sepsis rate was 10.3% (30/292), whereas 7 day and 30 day mortality in our cohort was 3.1% (9/292) and 16.1% (47/292), respectively. The levels of bilirubin at day 7 had decreased in 74% (216/292) with 57% (167/292) remaining decreased after 30 days. Overall, in the majority of cases (91%) there were no immediate complications. The most common immediate complication was post-operative pain (8.2%), followed by 2 cases of stent dislodging and 3 cases of pancreatitis and one case of haemorrhage that was not life-threatening. There was no significant difference when comparing the above in the malignant versus the non-malignant groups.

### Subgroup analysis

Modified Karnofsky (Table 3).

**Table 3:** Outcomes in high vs low risk modified Karnofsky score

	Modified Karnofsky, High risk	Modified Karnofsky, Low risk	*P*
7d Mortality (%)	9.7	0	0.002
30d Mortality (%)	28.8	13.3	0.003
30d Sepsis (%)	19	3.3	0.001
Bilirubin decreased at 7d(%)	76.3	76.5	0.8
Bilirubin decreased at 30d (%)	70.5	65.1	0.6

In the modified Karnofsky high risk group (5 +) the 7 day mortality was 9.7% versus 0% for the low risk group (Pearson Chi square 9.6;df: 1; *p* = 0.002), whereas the 30 day mortality was 28.8% versus 13.3% (Pearson Chi square 5.7;df:1; *p* < 0.05). The incidence of sepsis at 30 days was 19% in the high risk group versus 3.3% at the low risk group (Pearson Chi square 10.5; df:1; *p* = 0.001). Bilirubin levels in day 7 decreased in 76.5% of the low risk group and in 76.3% in the high risk group without a statistically significant difference (*p* = 0.8) and this trend was continued at day 30 (65.1% vs.70.5%, *p* = 0.6). There was no significant difference when comparing the above in the malignant versus the non-malignant groups.

### Standard Karnofsky Score

**Fig. 1:**
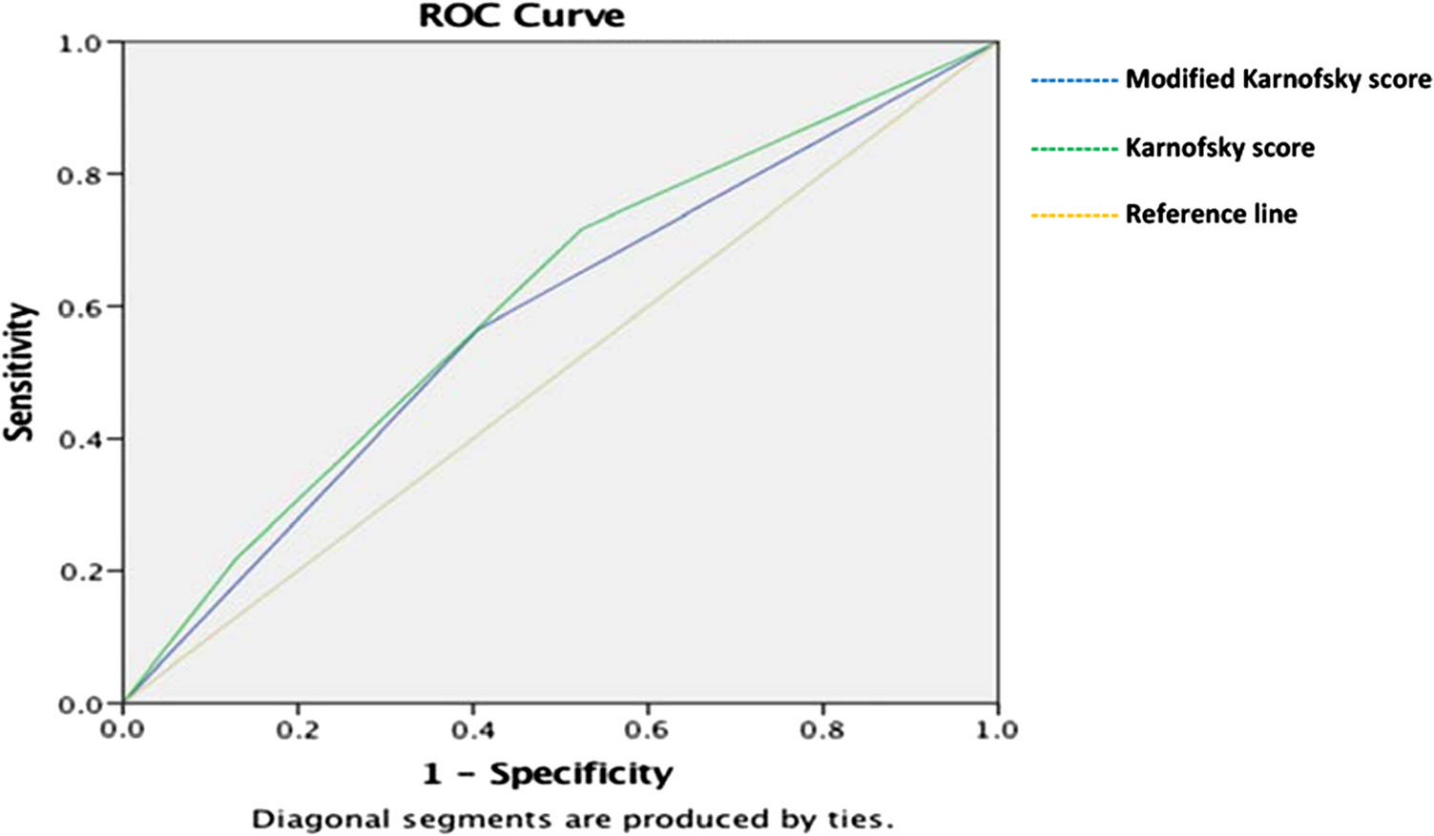
30 day mortality ROC curves between the two performance scores

**Fig. 2:**
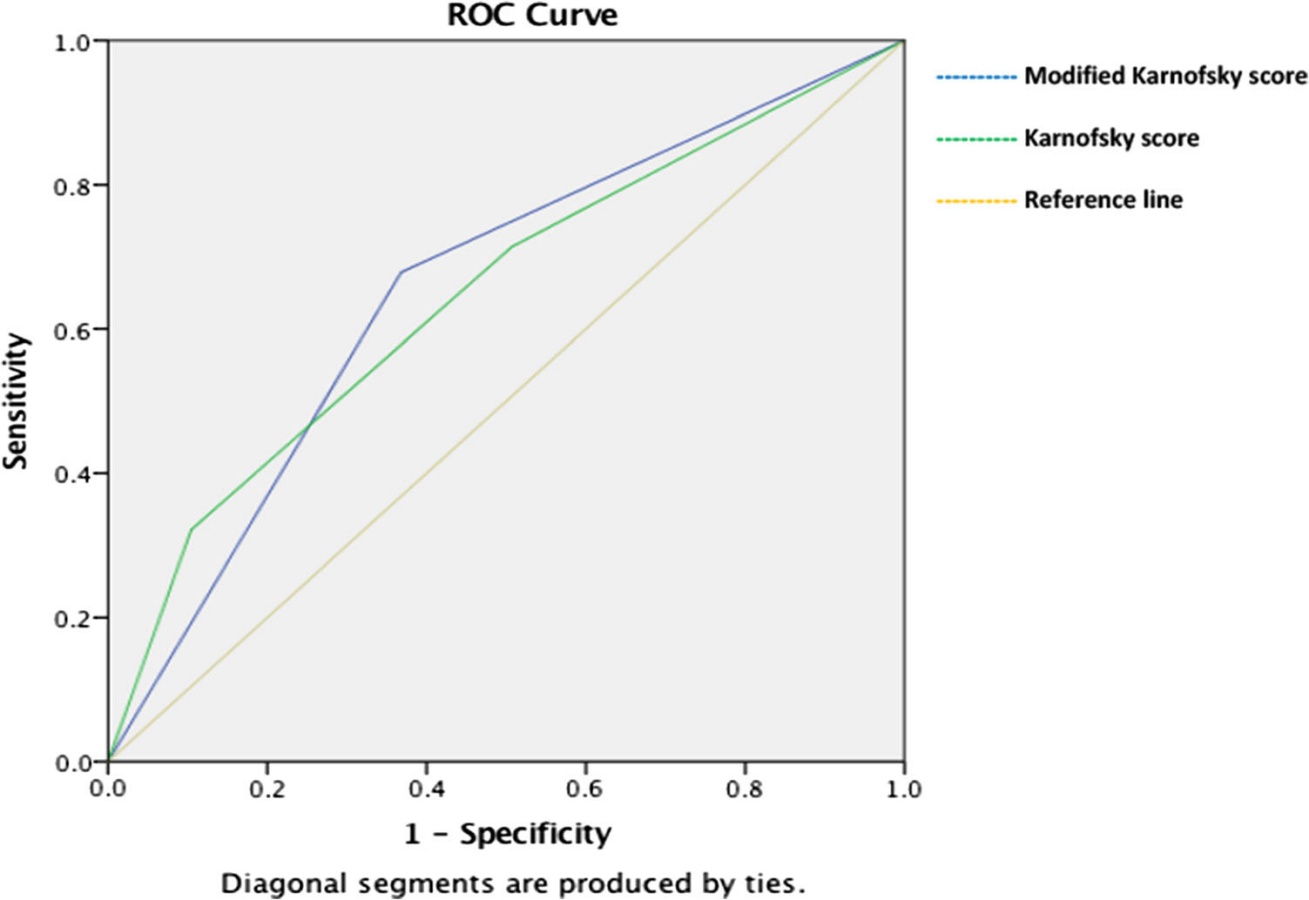
7 day sepsis ROC curves between the two performance scores

When we assessed sepsis and mortality using only the Karnofsky score for risk stratification, there was also a statistically significant difference in sepsis at day 30 (Pearson Chi-Square:15.897,df: 6; *P* = 0.014) and 30-day mortality (Pearson Chi-Square;14.882,df 6;P = 0.021) with higher mortality and sepsis rates in the group with Karnofksy score of < 60. When the two scores were compared using ROC curves for the 30 day mortality the modified Karnofsky score had an area under the curve of 0.580 (SE:0.47 with 95%CI:0.488–0.671) versus 0.607 (SE0.46 with 95%CI 0.518–0.696) for the Karnofsky score without any statistically significant difference (Fig. 1). The same trend was noted for 30 day sepsis with the modified Karnofsky score having an area under the curve of 0.655 (SE:0.55 with 95%CI:0.548–0.763) versus 0.648 (SE0.59 with 95%CI 0.533–0.763) for the Karnofsky score without any statistically significant difference (Fig. 2).

Primary Stenting vs Staged Procedure (Tables 4 and 5).

**Table 4:** Outcomes in stented vs drain only subgroups

	Stented	Drain only	*P*
7d Mortality (%)	5.8	1.6	0.1
30d Mortality (%)	22.1	18	0.5
30d Sepsis (%)	11.7	8.3	0.5
Bilirubin decreased at 7d (%)	79.3	72.7	0.46
Bilirubin decreased at 30d (%)	69.7	62.1	0.3

**Table 5:** Outcomes following one or two stage stent procedures

	Two stage stent	One stage stent	*P*
30d Mortality (%)	15	19.2	0.194
30d Sepsis (%)	7.8	11.7	0.197

The incidence of sepsis at day 30 was higher at the stented group, 11.7% versus 8.3% in the drain-only group (*p* = 0.5), however this difference did not reach statistical significance. Regarding mortality after 7 and 30 days in the stented versus the drain-only group, again, there was a trend for higher mortality in the stented group (Day 7: 5.8% versus 1.6%, *p* = 0.1 and Day 30: 22.1% versus 18%, *p* = 0.5) but without reaching statistical significance. The 30 day sepsis rate was 7.8% for the group that had stenting as a two stage procedure versus 11.7% for the single stage stenting group, however the difference was not statistically significant (Pearson 6.027, df:4; *p* = 0.197).

In the subgroup analysis using the modified Karnofsky score, the incidence of sepsis between the high and low risk groups in the drain only cases was not statistically significant (15.4% versus 7.5%, *p* = 0.444), but this difference was significant in the stented group. More specifically, the 30 day sepsis, in the drain-only patients was 13% for high risk versus 5.4% for low risk, *p* = 0.2 , while in the stent group it was 23.1% for high risk versus 1.9% for low risk, *p* = 0.001. The 7 and 30 day mortality for the stent versus drain-only patients was 13.3% versus 3.8%, *p* = 0.19 and 27.5% versus 32%, *p* = 0.69, respectively.

In the binary regression model the high risk group according to modified Karnofsky score (*p* = 0.035), the type of stricture (*p* = 0.019) and the level of bilirubin postoperatively (*P* = 0.004) appeared to be significantly associated with the incidence of overall mortality at 30 days (*P* = 0.035). Regarding the incidence of sepsis at 30 days, female gender (*P* = 0.046), use of antiplatelets (*p* = 0.017) and the being at the high risk group of the modified Karnofsky score (*p* = 0.015) were the only factors that appeared to be significantly associated with increased risk of sepsis. When the binary regression model was adjusted to assess only the malignant lesions in the cohort, the high risk group according to the modified Karnofksy score remained a significant factor (p < 0.05) along with level or postoperative bilirubin (*p* < 0.05).

## Discussion

This is the first study to our knowledge to prospectively assess a scoring system in predicting the outcomes of biliary drainage and stenting procedures. Patients undergoing biliary procedures are generally unwell and our study supports the notion that the patient’s clinical status is a key determinant in affecting mortality and morbidity post PTBD. Indeed our study showed that high risk patients in the modified Karnofsky score show worse outcomes when it comes to mortality and sepsis incidence. In addition, although the choice between stent or drain insertion did not seem to affect mortality and morbidity, a difference was found on subgroup analysis of the Modified Karnofsky high-risk group versus the low risk groups, with high risk patients who received stents appearing to have worse outcomes than those who were in the low risk groups. The fact that patients in the high risk clinical group appear to be doing worse when receiving biliary stents than those receiving drains only may be related to increased procedural time and increased risk of bacteraemia which in combination with the poor performance status could explain the poor outcomes. However, despite these important findings, both the Karnofsky and modified Karnofsky scores, did not show high differentiation ROC curves for mortality and morbidity and were not shown to be significantly different from each other which might be a result of the relatively small sample size.

We had previously carried out a small local pilot study in 50 consecutive patients and based on the positive finding in this cohort wanted to test the modified Karnovsky scoring system in a larger cohort in a multi-centre setting. The modified Karnofksy score incorporates a number of significant biochemical factors to the pre-existing clinical score which have previously been shown to independently correlate with worse outcomes and therefore cannot be disregarded without further research. When assessing the technical factors that might be affecting mortality and morbidity, it was noted that in the stent only group there was a trend for higher incidence of sepsis at 30 days and higher 30 day mortality, however this was not statistically significant. When assessing one versus two-stage stenting affects mortality and morbidity, the trend for sepsis and 30 day mortality were lower in the group that had a two-stage procedure, however, these results also did not reach statistical significance. Furthermore, our study showed no statistically significant difference in mortality or morbidity when comparing patients who received stent versus those who only had a drain. Regarding the issue of performing the procedure in one or two-stages, there is limited evidence from previous studies which suggests a two-stage procedure does not have any clear benefits on mortality and morbidity but associated with increased the cost and procedure time. [11–14]. It does seems however that centre experience may also be an important factor and those centres that performs more than 28 procedures per year have a significantly lower mortality [15].

This is the largest prospective study in the United Kingdome and confirms that PTC is a technically feasible and effective procedure (95% technical success rate) with similar mortality and morbidity rates as reported elsewhere in the literature [16]. Despite the majority of our study participants having a relatively good performance status, the incidence of 30-day sepsis was 10% and the overall 30 day mortality was 16%. These results reaffirm the findings of other large series [4, 15].

This study has limitations that should be taken into account. Data quality and completeness are often significant concern, since it represents a prospective, voluntary data collection effort from various centres and physicians across the UK and could be a non-consecutive patient cohort unrepresentative of the entire treated population. We did however ensure optimal data collection in this study with follow up contacts with all centres to achieve data completeness. Another potential limitation comes from the fact that participating clinician was given the freedom to select patients on local criteria and to treat them using their local protocols, which increased the heterogeneity of observed treatment and follow-up practices. However this did allow us to ensure that we were testing the scoring systems in a real-world scenario.

## Conclusion

PTB plays a key role in managing patients with biliary obstructions who are often severely debilitated and at high risk. However technical success rates remain excellent with a good safety profile. Scoring systems such as the Karnofsky or the modified Karnofsky score potentially allow us to identify high risk groups that will need more careful consideration, risk factor modification and enhanced patient informed consent. Further research with larger studies is warranted in order to identify their true impact on patient selection and outcomes post biliary interventions.
